# Social Anxiety Profiles and Psychopathological Symptom Differences in Spanish Adolescents

**DOI:** 10.1007/s10578-024-01756-5

**Published:** 2024-09-09

**Authors:** Dori J. A. Urbán, José M. García-Fernández, Candido J. Ingles

**Affiliations:** 1 Department of Developmental Psychology and Didactics, University of Alicante, Alicante, Spain; 2 Department of Health Psychology, Miguel Hernandez University of Elche, Avda. de la Universidad, s/n. 03202, Elche (Alicante), Spain

**Keywords:** Latent profile analysis, Psychopathological symptoms, Social anxiety, Adolescence

## Abstract

Research on social anxiety (SA) over the years has revealed its associations with different psychopathological symptoms. This study aims to characterize SA profiles in a sample of Spanish adolescents and explore their differences in psychopathological symptoms. Data from 1,288 Spanish students in the 15 to 18 age range (*M* = 16.30, *SD* = 0.97, 47.5% female) were collected using random cluster sampling. The *Social Anxiety Scale for Adolescents* (SAS-A) and the *Symptom Assessment-45 Questionnaire* (SA-45) were employed. Four SA profiles were revealed by the Latent Profile Analysis (LPA): *extreme SA*,* high SA*,* moderate SA*, and *low SA*. Statistically significant differences in psychopathological symptoms were revealed by the MANOVA (effect sizes from *d* = -2.13 to *d* = -0.37). The *extreme SA* profile exhibited the most severe psychopathological symptoms, whereas the *low SA* profile displayed the mildest manifestations. Proposed interventions aim to support adolescents with SA risk profiles.

## Introduction

Social anxiety (SA) involves an intense fear of social situations and the expectation of being evaluated by others [13, 46]. Typically emerging during adolescence, adolescents are particularly vulnerable to SA during this critical developmental stage marked by significant biological, psychological, and social changes.

According to Inglés et al. [25], the prevalence of social anxiety was 12.06% in a community sample of Spanish adolescents, with rates of 8.80% in boys and 15.47% in girls. More recent data indicate a significant increase in prevalence, now reported to be 29.7% [10]. Additionally, prevalence rates can vary widely depending on the self-report measures used and the specific country in which the studies are conducted [26].

The presence of comorbid conditions can exacerbate the severity of SA, leading to significant interference in daily functioning and social adaptation. The onset of SA is influenced by various factors including psychological vulnerability and social stressors. The developmental transition from middle to late adolescence is a critical period where individuals are more susceptible to the emergence of SA and other psychiatric disorders [29]. The heightened sensitivity to peer evaluation and the significant social changes experienced during this time contribute to the initial development of SA [4].

Social anxiety is not a uniform condition but rather a spectrum of experiences that can vary widely across individuals. Social anxiety is often characterized by three principal constructs as described by La Greca and López [28]: fear of negative evaluation (FNE), social avoidance and distress-general (SAD-G), and social avoidance and distress-new (SAD-N). Fear of negative evaluation (FNE) involves the apprehension about being judged or negatively evaluated by others, which can lead to significant emotional distress and interpretation biases in social contexts (Dapprich et al., [8]; Delgado et al., [10]. Social avoidance and distress-general (SAD-G) reflects a general tendency to avoid social interactions and experience distress across various social contexts. Social avoidance and distress-new (SAD-N) pertains to the discomfort and avoidance experienced in new or unfamiliar social situations. These constructs provide a comprehensive framework for understanding the multifaceted nature of SA and its impact on adolescents.

However cross-cultural differences must be taken into account. The analyses of latent differences between community samples in Spain, China and the USA, as conducted by Torregrosa et al. [44], revealed that Spanish adolescents scored significantly higher than their North American peers on FNE (TS = -9.630; *d* = 0.44) and SAD-G (TS = -2.717; *d* = 0.12), and that the perception of social anxiety varies between China and Western countries. Consequently, cultural values and social norms affect how social anxiety symptoms manifest and their severity [33, 40]; Torregrosa et al., 2022, [44]; Zhou et al., [50].

In addition to social anxiety, it is important to consider the broader dimensions of psychopathology that frequently coexist with SA. These dimensions include depressive symptoms, generalized anxiety, and behavioral issues, which often interact with SA to affect overall mental health (Newman et al., [35]). For instance, depressive symptoms can exacerbate the severity of SA by increasing feelings of worthlessness and hopelessness [23], while generalized anxiety can overlap with SA, leading to heightened overall anxiety levels. Behavioral problems, such as aggression or conduct disorders, can also compound the difficulties associated with SA. Understanding these dimensions in the context of SA is critical for developing a comprehensive approach to intervention and treatment.

### Research on Social Anxiety Profiles

An emerging area of research focuses on the concept of social anxiety profiles, which refer to distinct patterns or clusters of social anxiety symptoms that individuals may exhibit. These profiles can provide a more nuanced understanding of how social anxiety manifests and varies among individuals. Social anxiety profiles are categorized based on different dimensions of social anxiety, such as fear of negative evaluation, social avoidance, and distress in various social contexts. Identifying and understanding these profiles can help in tailoring interventions to address specific patterns of social anxiety more effectively.

The investigation into social anxiety profiles is crucial because it allows for the identification of distinct subgroups within the adolescent population who may experience social anxiety differently. For instance, some adolescents may exhibit high levels of fear of negative evaluation but low levels of general social avoidance, while others may show the opposite pattern. This differentiation helps in understanding the heterogeneous nature of social anxiety and how it relates to broader psychopathological symptoms.

Once established, SA can be maintained by several factors. Adolescents with SA often engage in avoidance behaviors to evade distressing social situations, which can reinforce and maintain their anxiety [17]. Additionally, cognitive processes such as rumination and anticipatory worry can perpetuate SA by continually focusing attention on potential negative outcomes and perceived inadequacies [32]. The impact of SA on adolescents is profound, affecting their academic performance, peer relationships, and overall quality of life (Ranta et al., 2009 [38]; Vilaplana-Pérez et al., [47]). Adolescents with SA are more likely to experience social withdrawal, loneliness, and lower self-esteem, which can hinder their social and emotional development (La Greca & Lopez, [28]). Furthermore, the avoidance behaviors associated with SA can lead to missed educational and social opportunities, perpetuating a cycle of social isolation and anxiety (Aderka et al., 1). These maintenance factors contribute to the persistence of SA and hinder the development of effective coping strategies.

The consequences of SA impact several aspects of adolescent life. Social anxiety can lead to significant impairments in academic performances and interpersonal relationships, as individuals may fear displaying symptoms that might result in negative evaluations or rejection [19, 45, 47]. This often results in increased loneliness [27] and compromised friendship quality, although the latter can sometimes serve as a protective factor [5]. The long-term impact of SA is considerable, with early experiences of SA being associated with subsequent internalizing disorders and a higher likelihood of developing social anxiety disorder (SAD) [22] in late childhood or adolescence (Wong & Rappe, [48]; Liebowitz et al., [30]). SAD is one of the most prevalent mental disorders in adolescence and affects approximately 8.3% of adolescents [13, 40].

In a broader context, experiencing SA in early development may be a key factor leading to subsequent internalizing disorders in adolescence and often anticipates the diagnosis of social anxiety disorder (SAD) [22]. SAD is mostly diagnosed during late childhood and adolescence (Wong & Rappe, [48]).

Social anxiety (SA) is a prevalent and debilitating condition among adolescents, often co-occurring with other psychopathological symptoms such as depression, generalized anxiety, and behavioral problems. Understanding the nuanced relationships between SA and these co-occurring symptoms is critical for developing targeted interventions and improving mental health outcomes in this population. Investigating the co-occurrence of SA and other psychopathological symptoms in adolescents aged 15 to 18 is essential, as this age range encompasses key transitions, such as the move from middle to late adolescence, which can influence mental health trajectories.

Traditional approaches to studying SA and its comorbidities often rely on variable-centered methods, which may obscure the heterogeneity within the adolescent population. These methods assume homogeneity within the population, potentially overlooking distinct subgroups that may exhibit unique patterns of symptomatology and require tailored therapeutic approaches. Socially anxious individuals often manifest additional psychopathological symptoms, and the majority of those with SAD experience comorbid mental disorders (Liebowitz et al., [30]). Therefore, it is crucial to explore the relationship between SA and psychopathological symptoms, as subclinical SA could potentially shed light on the increased severity of psychopathological symptoms in adolescents and its potential implications for the current and future development of mental disorders. Research at the subclinical level is essential for early identification and intervention, as many adolescents may not meet the criteria for clinical diagnoses but still experience significant distress and impairment.

### The Present Study

In this study we employed the *Social Anxiety Scale for Adolescents* (SAS-A) from La Greca and López [28] and the *Symptom Assessment-45 Questionnaire* (SA-45), developed by Davison et al. [9]. While initially designed for adults, the SA-90-TR, predecessor of the SA-45, had been used in prior research with adolescents. However, due to its length and complexity, the SA-45 was considered for our study because of its broad scope and prior use with adolescents aged 15 to 18 years in Spain (Fernández-Sogorb, [14], Gonzálvez et al., [18]), supporting the reliability and validity of our findings.

While it is known that comorbidity between different forms of psychopathology is common, our study provides a detailed latent profile analysis (LPA) specifically focusing on social anxiety profiles among Spanish adolescents. This demographic has been less frequently studied, particularly using LPA, which allows for the identification of distinct subgroups within the population that may not be apparent through other analytical methods. This study offers a nuanced understanding of how different profiles of social anxiety correlate with a broader range of psychiatric symptoms, including various dimensions of psychopathology. This has practical implications for targeted interventions. There is a significant need for more sophisticated data analyses to examine the co-occurrence of SA and psychopathological symptoms in the 15 to 18 age range. To address this existing void, Spanish adolescent subgroups were detected through latent profile analysis (LPA). LPA is a person-centered statistical technique that allows us to identify heterogeneity within a population based on individuals’ responses to multiple indicators, offering insights into distinct patterns of social anxiety that could provide a more nuanced understanding of how SA manifests alongside other psychopathological symptoms, and inform tailored therapeutic approaches that address the specific needs of each subgroup.

This study aims to explore the specific profiles of social anxiety (SA) and their association with a range of psychopathological symptoms. By focusing on SA symptoms, we aim to identify distinct subgroups within the Spanish adolescent population that are particularly relevant to the manifestation and treatment of social anxiety. We also hypothesize that there will be four distinct social anxiety profiles among Spanish adolescents grounded on prior research.

The general objective of the current research is to examine whether there are statistically significant differences in psychopathological symptoms among different SA profiles in Spanish adolescents. The specific objectives are: (1) To identify the number of SA profiles in Spanish adolescents; (2) To establish the differences in psychopathological symptoms across the SA latent profiles. Our hypotheses are as follows: Hypothesis 1: Four SA profiles are expected to emerge among Spanish adolescents. Hypothesis 2: Greater levels of SA are expected to be linked to greater scores in psychopathological symptoms.

## Methods

### Participants

Participants were selected through a random cluster sampling approach. Ten secondary schools from the province of Alicante in southeastern Spain were randomly selected. The initial sample consisted of 1,343 participants, from which 11 adolescents (0.82%) were excluded due to an inadequate command of Spanish. Additionally, 37 adolescents (2.75%) were excluded for not providing written informed consent, and 7 adolescents (0.52%) voluntarily withdrew from the study.

Participants willingly took part in the current study, and they were free to withdraw at any point and received no incentives. Test administration was conducted using Google Forms to ensure efficient data collection, to avoid order bias, and to minimize the risk of outliers and missing data, which were eliminated before performing the statistical analyses.

The sample of the study included 1,288 adolescents (52.5% male, 47.5% female), all aged between 15 and 18 (refer to Table 1). The average age was 16.30, with a standard deviation of 0.97.

**Table 1:** Participants sample distribution by age and gender

Age Groups and Gender	15 years *n* %	16 years *n* %	17 years *n* %	18 years *n* %	Total *N* %
Male	154 12.0%	250 19.4%	185 14.4%	87 6.8%	676 52.5%
Female	158 12.3%	189 14.7%	188 14.6%	77 6.0%	612 47.5%
Total	312 24.2%	439 34.1%	373 29.0%	164 12.7%	1288 100.0%

A test of homogeneity using Pearson’s chi-square test was performed to assess the distribution of gender and age groups. The analysis involved eight groups, each representing a combination of two genders within four age groups. No statistically significant differences were detected among the eight Gender x Age groups (χ^2^ = 5.99, *p* = .112).

### Instruments

The *Social Anxiety Scale for Adolescents* (SAS-A; La Greca & López, [28]) is designed to assess adolescents’ self-perception of SA. It is composed of 18 self-report items and 4 filler items, organized into three subscales. The *fear of negative evaluation* (FNE) subscale has 8 items addressing concerns about peer negative evaluations. The *social avoidance and distress-general* (SAD-G) subscale includes 4 items related to general social inhibition, discomfort and distress. The *social avoidance and distress-new* (SAD-N) subscale involves 6 items on social avoidance and distress in new or unfamiliar social situations: Participants use a five-point Likert-type scale, where 1 corresponds to “not at all” and 5 represents “all the time”. The levels of SA are represented by the total SAS-A score (18–90). Higher scores indicate greater SA. The scores above 44 reveal SA, whereas scores below 37 suggest a lack of SA [36].

The SAS-A has undergone adaptation in several countries, such as Spain [16], Portugal [7], and China [50]. In the current study, the Spanish adaptation of the SAS-A [16] was used. It has high internal consistency, with Cronbach’s α = 0.94 for FNE, α = 0.80 for SAD-G, α = 0.87 for SAD-N, and α = 0.91 for SAS-A total score. In this study, the reliability coefficients for the SAS-A subscales were Cronbach’s α = 0.85 for FNE, α = 0.78 for SAD-G, and α = 0.74 for SAD-N; and McDonald’s ω = 0.85 for FNE, ω = 0.78 for SAD-G, and ω = 0.74 for SAD-N.

The *Symptom Assessment-45 Questionnaire* (SA-45; Davison et al., [9]) consists of 45 items designed to assess psychopathological symptoms. Adapted from the original SCL-90, it consists of nine five-item scales: *anxiety*,* depression*,* hostility*,* interpersonal sensitivity*,* obsession-compulsion*,* paranoid ideation*,* phobic anxiety*,* psychoticism*, and *somatization*. For this study, the SA-45 Spanish version, validated for Spanish students [41], was used. Participants report the frequency of the 45 psychopathological symptoms over the preceding week using a five-point Likert scale, where 0 corresponds to “not at all” and 4 represents “very much” or “extremely.” The reliability coefficients for the SA-45 subscales were as follows: Cronbach’s α = 0.75 for *depression*, α = 0.80 for *hostility*, α = 0.77 for *interpersonal sensitivity*, α = 0.79 for *somatization*, α = 0.77 for *anxiety*, α = 0.70 for *psychoticism*, α = 0.73 for *obsession-compulsion*, α = 0.80 for *phobic anxiety*, and α = 0.72 for *paranoid ideation*; and McDonald’s ω = 0.76 for *depression*, ω = 0.80 for *hostility*, ω = 0.77 for *interpersonal sensitivity*, ω = 0.79 for *somatization*, ω = 0.77 for *anxiety*, ω = 0.71 for *psychoticism*, ω = 0.74 for *obsession-compulsion*, ω = 0.80 for *phobic anxiety*, and ω = 0.72 for *paranoid ideation*. For reference, previous studies with Spanish population have reported high internal consistency (e.g. Sandín et al., [41]).

### Procedures

First, we contacted the principals of each secondary school and explained the nature and purpose of this study to seek their collaboration. After informing the students that participation was voluntary and they had the option to withdraw at any point, the participants were required to submit the written informed consent from their parents or legal guardians during the following two weeks. The questionnaires were administered in the classroom during school hours, through Google Forms to preserve anonymity, ensuring data collection without associating the responses with the specific adolescents. Additionally, the counterbalancing procedure was implemented using Google Forms to avoid the bias related to the order effect in instrument administration. On average, the administration of instruments required 45 min. In every classroom, there was a researcher who delivered the instructions, addressed concerns, and ensured participants completed the scales on their own.

Ethical standards were followed in this study, aligning with the Declaration of Helsinki of 1964, and the International Ethical Code in Humanities and Social Sciences. It also received the approval of the Ethics Committee of the University of Alicante under protocol number UA-2019-07-10.

### Data Analysis

Initially, this study examined the connections among the dimensions of the *Social Anxiety Scale for Adolescents* (SAS-A) and the *Symptom Assessment-45 Questionnaire* (SA-45) using Pearson correlation coefficients. Interpretation followed Cohen’s [6] criteria, categorizing correlations as follows: 0.10–0.29 as low correlation, 0.30–0.49 as moderate correlation, and 0.50 or higher as strong correlation.

To identify the best-fit model, an LPA was conducted, comparing models ranging from 2 to 8 profiles. Each profile model was defined based on standardized scores from the three SAS-A subscales: Fear of Negative Evaluation (FNE), Social Avoidance and Distress-General (SAD-G), and Social Avoidance and Distress-New (SAD-N). Before LPA, standardization of scores from the three SAS-A factors was done, with z-scores below − 0.5 denoting low SA, -0.5 to 0.5 indicating moderate SA levels, and over 0.5 reflecting high SA.

For determining the final profile solution, various factors, including multiple fit values, content decision criteria [43], and indices were considered. The optimal model required lower values of Akaike Information Criteria (AIC) and Low Bayesian Information Criteria (BIC); *p* ≤ .05 for the Bootstrap Likelihood Ratio Test (BLRT) and the Vuong-Lo-Mendell-Rubin Likelihood Ratio Test (LRT), and an entropy score nearing 1. Subgroups were required to include a minimum of 1% of the sample. After establishing the best-fit model, the χ^2^ statistical was used to check if there was a similar distribution of female and male adolescents in each cluster.

After the profile identification, a MANOVA was conducted to examine potential variations in the mean scores of psychopathological symptoms among the profiles. The conditions of normality and homoscedasticity were met. The interpretation of effect sizes was based on Cohen’s *d*-values [6], where values from 0.20 to 0.49 denoted small, from 0.50 to 0.79 indicated medium, and over 0.80 represented large effect sizes. The data analysis was conducted using MPlus version 8 software (Muthén & Muthén, 2017) and SPSS version 26 [24].

## Results

Regarding the correlations among SAS-A and SA-45 factors, FNE shows a moderate correlation with *depression*,* hostility*,* somatization*,* psychoticism*,* obsession-compulsion* and *paranoid ideation*, and high correlation with *interpersonal sensitivity*,* anxiety* and *phobic anxiety.* As for SAD-G and SAD-N, there is a moderate correlation with all SA-45 factors. Detailed correlations are provided in Table 2.

**Table 2:** Correlations between SAS-A and SA-45 factors

Variable	FNE	SAD-*N*	SAD-G
depression	0.474**	0.405**	0.438**
hostility	0.413**	0.306**	0.339**
interpersonal sensitivity	0.534**	0.434**	0.474**
somatization	0.413**	0.353**	0.394**
anxiety	0.510**	0.433**	0.471**
psychoticism	0.469**	0.360**	0.387**
obsession-compulsion	0.447**	0.423**	0.420**
phobic anxiety	0.522**	0.446**	0.476**
paranoid ideation	0.493**	0.401**	0.434**

*Note* FNE: fear of negative evaluation; SAD-G: social avoidance and distress-general; SAD-N: social avoidance and distress-new **p < .001

In this study, LPA is employed to identify different subgroups of Spanish adolescents by evaluating their scores on the *Social Anxiety Scale for Adolescents* (SAS-A). The selection of the best-fit model was guided by both statistical criteria and the interpretability of profiles. Table 3 presents the goodness-of-fit indices for the estimated models.

**Table 3:** Adjustment indices for latent profile models of SA and psychopathological symptoms

Models	AIC	BIC	BIC-adjusted	LRT *p*	LRT-adjusted	BLRT	Entropy	SIZE
2	9551.23	9602.83	9571.07	< 0.001	< 0.001	< 0.001	0.82	0
3	9054.97	9127.23	9082.75	< 0.001	< 0.001	< 0.001	0.88	0
**4**	**8895.34**	**8988.24**	**8931.06**	**< 0.001**	**< 0.001**	**< 0.001**	**0.78**	**0**
5	8819.68	8933.22	8863.34	0.052	0.068	< 0.001	0.79	0
6	8787.49	8921.68	8839.09	0.184	0.197	< 0.001	0.73	0
7	8770.81	8925.64	8830.34	0.478	0.487	< 0.001	0.76	1
8	8726.73	8902.20	8794.19	0.720	0.727	< 0.001	0.76	0

*Note* AIC: Akaike Information Criteria; BIC: Bayesian Information Criteria; LRT: Vuong-Lo-Mendell-Rubin Likelihood-Ratio Test; BLRT: Bootstrap Likelihood Ratio Test; SIZE = Number of clusters with fewer than 25 subjects or 1% of subjects size. Fit criteria indicating the best model are printed in bold

The models with five to eight profiles have lower AIC and BIC values, along with entropy values of 0.7. However, they are rejected because the LRT *p*-value is above 0.05, signaling a lack of statistical significance, and the seven-profile model includes a subgroup representing less than 1% of the sample, making it less representative and less meaningful.

Regarding the two, three and four-profile models, the best fit one is the four-profile one as it meets the preferential criterion of having the lowest BIC and AIC. Despite not having the highest entropy index (0.78), each subgroup within the four-profile model is well-represented in the sample, and the four-profile model still exhibits statistical significance with a *p*-value less than 0.05 for the BLRT and the LRT, offering a better fit in comparison with other models.

Our selected solution categorized SA into four distinct profiles: *low SA* (26.6%), *moderate SA* (38.1%), *high SA* (31.2%) and *extreme SA* (4.1%). Adolescents with *low SA* (*n* = 342) had no clinically significant scores across all three dimensions of SA, exhibiting minimal-to-mild SA symptoms and remaining relatively functional, even though they could experience some nervousness in social contexts. Those with *moderate SA* (*n* = 491), the largest subgroup, had moderate scores in the three dimensions of the SAS-A experiencing symptoms such as embarrassment, avoidance of social situations and difficulties in interpersonal sensitivity. Adolescents with *high SA* (*n* = 402), the second largest subgroup, displayed high scores in all three dimensions of the SAS-A, with symptoms like panic attacks, physical symptoms and isolation. Lastly, adolescents with *extreme SA* (*n* = 53) scored very high in SA and might meet the criteria for SAD.

The chi-squared test results indicated similar gender (χ2 = 3.24, *p* = .36) and age (χ2 = 10.26, *p* = .33) group distributions across the identified latent profiles, with no significant differences observed (see Table 4).

**Table 4:** Distribution of gender and age across latent profiles

Profiles	Total	Gender		Age			
		Male	Female	15-year-olds	16-year-olds	17-year-olds	18-year-olds
		χ2 = 3.24, *p* = .36		χ2 = 10.26, *p* = .33			
low SA	342 (26.6%)	191 14.8%	151 11.7%	78 6.1%	128 9.9%	100 7.8%	36 2.8%
moderate SA	491 (38.1%)	245 19.0%	246 19.1%	118 9.2%	165 12.8%	134 10.4%	74 5.7%
high SA	402 (31.2%)	210 16.3%	192 14.9%	107 8.3%	130 10.1%	119 9.2%	46 3.6%
extreme SA	53 (4.1%)	30 2.3%	23 1.8%	9 0.7%	16 1.2%	20 1.6%	8 0.6%

*Note* SA = social anxiety

The aim of the MANOVA was to evaluate the mean scores of psychopathological symptoms across distinct SA profiles. The results, as shown in Table 5, revealed significant differences among the latent SA subgroups through all SA-45 dimensions, with Wilks’ lambda = 0.68, and (*F*_(27.1284)_ = 19.80, *p* < .001, n_p_^2^ = 0.12). A large *F* -value with an associated *p <* .001, suggests that there is a significant variation in one or more of the dependent variables among the subgroups.

**Table 5:** Multivariate contrasts^**a**^

Effect		Value	F	Degrees of freedom of the hypothesis	Degrees of freedom of the error	Sig.	Partial eta squared
SASA4L	Pillai’s trace	0.33	17.43	27.00	3834.00	0.000	0.11
	Wilks’ lambda	0.68	19.80	27.00	3727.22	0.000	0.12
	Hotelling’s trace	0.47	22.33	27.00	3824.00	0.000	0.13
	Roy’s largest root	0.46	65.30^c^	9.00	1278.00	0.000	0.32

a. Design: SASA4L

b. Exact statistic

c. The statistic is an upper limit for F, which provides a lower limit for the level of significance

The SA-45 factors showed significant differences among the four SA subgroups, as indicated in Table 6. As SA levels increase from low to extreme, the average psychopathological symptoms scores also increase. This implies that there is a positive relationship between SA and psychopathological symptoms in this study. Specifically, the *extreme SA* profile presented the highest mean scores across all dimensions of the SA-45, while the *low SA* profile had the lowest ones.

**Table 6:** Means, standard deviations, and statistical significance of SA-45 subscales across SA profiles

	low SA profile		moderate SA profile		high SA profile		extreme SA profile		Statistical Significance	
*Dimensions*	*M*	*SD*	*M*	*SD*	*M*	*SD*	*M*	*SD*	*F*_*(3,1284)*_	*P*
depression	3.96	3.61	6.13	3.76	8.51	4.13	11.56	4.94	114.89	< 0.001
hostility	4.51	4.27	6.08	4.13	8.43	4.27	10.32	4.99	68.10	< 0.001
interpersonal sensitivity	3.95	3.59	6.23	3.67	8.87	3.70	11.64	4.98	141.31	< 0.001
somatization	4.57	4.01	6.57	4.13	8.73	3.93	10.73	5.04	80.95	< 0.001
anxiety	3.69	3.65	6.15	3.61	8.72	3.77	11.07	4.55	141.29	< 0.001
psychoticism	3.44	3.34	5.50	3.72	7.45	3.61	9.58	4.94	94.69	< 0.001
obsession-compulsion	5.47	3.88	7.69	3.58	9.84	3.64	12.11	4.29	108.32	< 0.001
phobic anxiety	2.85	3.52	5.25	3.81	8.32	4.22	10.83	5.03	151.45	< 0.001
paranoid ideation	4.53	3.62	6.31	3.41	8.73	3.39	10.79	4.81	112.77	< 0.001

Table 7 presents the differences in SA-45 factor scores among the SAS-A profiles. The results indicate that there are significant variations in psychological symptoms across different levels of social anxiety. Specifically, profiles with higher social anxiety tend to have higher scores on depression, hostility, and other psychological factors compared to profiles with lower social anxiety. The most pronounced differences are observed between the low and extreme social anxiety profiles. Additionally, medium effect sizes are found between adjacent profiles, suggesting a gradient increase in symptom severity with higher social anxiety levels.

**Table 7:** Differences in SA-45 factor scores among SAS-A profiles

Dimensions		1–2 low SA vs. moderate SA	1–3 low SA vs. high SA	1–4 low SA vs. extreme SA	2–3 moderate SA vs. high SA	2–4 moderate SA vs. extreme SA	3–4 high SA vs. extreme SA
depression	*p*	< 0.001	< 0.001	< 0.001	< 0.001	< 0.001	< 0.001
	*d*	-0.59	-1.17	-1.99	-0.61	-1.40	-0.71
hostility	*p*	< 0.001	< 0.001	< 0.001	< 0.001	< 0.001	0.015
	*d*	-0.37	-0.93	-1.33	-0.56	-1.01	-0.4
interpersonal sensitivity	*p*	< 0.001	< 0.001	< 0.001	< 0.001	< 0.001	< 0.001
	*d*	-0.63	-1.35	-2.01	-0.72	-1.42	-0.72
somatization	*p*	< 0.001	< 0.001	< 0.001	< 0.001	< 0.001	< 0.001
	*d*	-0.49	-1.08	-1.48	-0.53	0.98	-0.49
anxiety	*p*	< 0.001	< 0.001	< 0.001	< 0.001	< 0.001	< 0.001
	*d*	-0.68	-1.35	-1.95	-0.70	-1.33	-0.61
psychoticism	*p*	< 0.001	< 0.001	< 0.001	< 0.001	< 0.001	< 0.001
	*d*	-0.58	-1.15	-1.71	-0.53	-1.06	-0.56
obsession-compulsion	*p*	< 0.001	< 0.001	< 0.001	< 0.001	< 0.001	< 0.001
	*d*	-0.60	-1.16	-1.69	-0.60	-1.21	-0.61
phobic anxiety	*p*	< 0.001	< 0.001	< 0.001	< 0.001	< 0.001	< 0.001
	*d*	-0.65	-1.40	-2.13	-0.77	-1.42	-0.58
paranoid ideation	*p*	< 0.001	< 0.001	< 0.001	< 0.001	< 0.001	< 0.001
	*d*	-0.51	-1.20	-1.65	-0.71	-1.26	-0.58

## Discussion

The objective of the current research was to examine whether there are statistically significant differences in psychopathological symptoms among different SA profiles in Spanish adolescents. With this objective in mind, LPA was conducted to classify homogeneous adolescent profiles displaying different SA patterns. This approach aims to promote timely detection and the implementation of preventive and corrective interventions for Spanish adolescents with distinct SA profiles to mitigate the adverse effects of SA on psychopathological symptoms in various spheres.

Using the SAS-A, four distinct subgroups were categorized, each representing different levels of SA. The findings support the first hypothesis, suggesting a four-profile solution, as four SA profiles emerged. These results align with prior literature [3, 11, 14, 42, 49]. Participants with excessive SA were categorized into the *extreme SA* profile, those with high SA into the *high SA* profile, moderate SA into the *moderate SA* profile, and low SA into the *low SA* profile. Given the efficacy of LPA in identifying homogeneous subgroups, further research on SA profiles involving LPA of SA in Spanish adolescents from different regions is necessary to validate these four subgroups.

In this study, the main difference among the SA profiles is the severity of psychopathological symptoms. Examining the differences among psychopathological symptoms within SA profiles reveals that the *extreme SA* subgroup scored higher in all psychopathological symptoms than the *high SA*,* moderate SA*, and *low SA* profiles. This outcome supports the second hypothesis, indicating that higher SA levels correspond to higher scores across multiple dimensions of psychopathological symptoms. Our findings suggest that adolescents within the *extreme SA* profile may experience a broad range of severe psychopathological symptoms and distress across various domains of functioning, especially in *obsession-compulsion*,* depression*,* interpersonal sensitivity* and *anxiety*. In contrast, adolescents within the *low SA* profile had the lowest scores in all dimensions of psychopathological symptoms, especially in *phobic anxiety*,* psychoticism* and *anxiety*.

These findings align with prior research (e.g. Forbes et al., [15, 21]. Specifically, Griffith et al. [20] observed a notable increase in the probability of SA and depression during adolescence. The study by Perino et al. [37] revealed, that higher social anxiety is linked to positive relations between irritability and aggression, although negative relations between irritability and perpetration [2, 12]. Similarly, Sanmartín et al. [42] observed that adolescents with a self-destructive profile scored very high in SA. Given these results, it is important to examine whether SA should be regarded as a thought disorder of higher-order, mainly inflexibility, which may contribute to the development of more severe disorders [22]. In addition, targeted interventions are suggested to enhance interpersonal functioning and provide a deeper understanding of SA. On-site interventions could focus on addressing SA and preventing stigma.

### Limitations

Despite the valuable insights provided by our cross-sectional study, several limitations must be acknowledged. The cross-sectional design restricts our ability to draw conclusions about causality or the temporal development of SA and associated symptoms. To address this, future research should employ longitudinal studies to explore the causal pathways and developmental trajectories of SA and its co-occurring symptoms. Additionally, this study relies on self-reported information, which may be subject to response biases such as social desirability, underscoring the need for careful interpretation of results derived from self-report questionnaires. Incorporating multi-method assessments, including clinical interviews, behavioral assessments, and physiological measures, would provide a more comprehensive evaluation of SA and other psychopathological symptoms. Additionally, while the Symptom Assessment 45 Questionnaire (SA-45) was chosen for its broad screening capabilities, it was originally developed for adults and has not been extensively validated with adolescents. Future studies should use adolescent-specific assessment tools to ensure accuracy and relevance. Moreover, the sample’s limitation to Spanish adolescents may affect the generalizability of the findings. Extrapolating these findings abroad is challenging owing to recognized intercultural differences in SA, shaped by diverse cultural norms and societal values, including individualism-collectivism [44]. Replicating this research in diverse cultural and demographic settings will help determine the universality of the identified SA profiles and their associated psychopathological symptoms.

The study does not distinguish between subclinical and clinical levels of SA, which could be an area for further exploration. Including measures for clinical case-ness could enable a more comprehensive understanding of how subclinical and clinical levels of SA and other psychiatric symptoms relate to each other. Additionally, longitudinal studies tracking individuals from subclinical to clinical levels of SA could provide insights into the development and risk factors associated with the escalation of symptoms. To further explore causality and the mechanisms underlying the observed associations, future research could benefit from employing experimental and quasi-experimental designs, which can help in testing the efficacy of targeted interventions and in understanding the causal pathways between SA and other psychopathological symptoms.

Additionally, examining differences among these classes based on demographic variables (e.g., age, gender, socio-economic status) or other collected variables (e.g., family background, academic performance) would provide a better understanding of how various background variables influence the manifestation and co-occurrence of SA and other psychiatric symptoms. Such an approach could lead to the development of tailored intervention strategies that are sensitive to the demographic and contextual nuances of different adolescent subgroups. Longitudinal studies would also be valuable in understanding the developmental trajectories of these latent classes and their long-term impact on adolescent mental health.

### Implications

In terms of implications for pediatric psychiatrists and clinical psychologists, the study underlines the importance of routine screenings for SA in adolescents. Validated tools like the SAS-A should be used to detect varying levels of SA, enabling early identification and intervention. Subclinical levels of SA can still significantly impact adolescents’ lives and may progress to clinical levels if left unaddressed. Personalized treatment strategies are crucial; for instance, adolescents with extreme SA might require more intensive therapeutic approaches compared to those with moderate or low SA. Tailoring interventions to address comorbid symptoms, such as combining cognitive-behavioral therapy (CBT) and pharmacotherapy for high SA with concurrent depressive symptoms or implementing behavioral interventions for high SA with concurrent behavioral problems, could enhance treatment effectiveness.

Holistic approaches are also recommended. Utilizing multi-method assessments and seeking interdisciplinary collaboration can improve overall care creating a supportive network for the adolescents, ensuring all aspects of their well-being are addressed. Practitioners should be trained to recognize and support adolescents with SA focusing on improving interpersonal skills and reducing social avoidance behaviors, while parents should be provided with resources to better support their children’s mental health. Culturally sensitive interventions are essential, as cultural and demographic factors may influence the manifestation of SA. Psychoeducating adolescents and their families about SA might help to reduce stigma and promote understanding, encouraging adolescents to seek help and participate actively in treatment.

Overall, these findings highlight the need for targeted, early interventions and ongoing support to address SA and its associated psychopathological symptoms effectively. Continued research and implementation of evidence-based practices are vital for improving outcomes for adolescents struggling with SA. Ensuring regular follow-up with adolescents to monitor their progress and adjust treatment plans as necessary, and providing long-term care, can help in identifying any emerging issues early and modifying interventions accordingly. Preventive strategies can be implemented in schools and communities to reduce the onset of SA and related psychopathological symptoms. Keeping up-to-date with the latest research on SA and related psychopathological conditions in adolescents can help psychiatrists and psychologists implement the most effective and evidence-based practices and developing better treatment strategies. This can include collaborating on longitudinal studies or experimental designs that explore the associations and mechanisms underlying SA and its comorbidities.

There are several implications for practice such as the design and implementation of preventive programs in educational settings to identify and support adolescents at risk of developing SA; prioritizing early intervention strategies to address SA and associated symptoms before they escalate into more severe disorders; and targeting interventions not only to reduce SA symptoms but also to improve overall functional outcomes, such as academic performance and social relationships. To achieve this, it is necessary to ensure that mental health practitioners receive training in the latest therapeutic techniques and approaches for treating SA and comorbid conditions, and that supervision and support for practitioners working with high-risk adolescent populations are provided to ensure effective treatment delivery and professional well-being.

Psychologists may implement classroom-based interventions that promote social skills and reduce anxiety, incorporating activities that foster a supportive and inclusive environment. Training for teachers should be provided to recognize signs of SA and other psychopathological symptoms. Educators should be equipped with strategies to support students and refer them to appropriate mental health resources. Furthermore, parental involvement should be encouraged by providing resources to help parents understand and support their children’s mental health needs.

The identification of distinct latent profiles based on SA symptoms and their associated psychopathological characteristics can inform the development of targeted intervention strategies and highlight the importance of early detection and treatment. Therefore, it is necessary to conduct preventive screenings and implement early on-site interventions to identify adolescents prone to SA and prevent the progression of further psychopathological symptoms. Identifying latent profiles of SA and comorbid symptoms can inform the development of personalized intervention strategies. For example, an adolescent with high SA and concurrent depressive symptoms may benefit from different therapeutic approaches compared to one with high SA and behavioral problems. Understanding these profiles can also help in early identification and prevention efforts, as well as guide clinicians in tailoring treatments to better address the complex needs of adolescents with multiple psychopathological symptoms. For instance, the outcomes of the study by Rao et al. [39] suggest that, for a subgroup of adolescents with SA, cognitive interventions could be suitable. Additionally, the results of the study conducted by Leigh et al. [31] on the efficacy of a variant of the Cool Kids anxiety program adapted to SA showed positive and significant results. Future research should consider the replicability of this study and explore whether the existence of psychopathological symptoms would also affect the results of the suggested interventions [27].

## Data Availability

No datasets were generated or analysed during the current study.
